# 2564. Population Pharmacokinetics and Pharmacodynamics of Cefepime in Hospitalized Obese and Non-Obese Patients

**DOI:** 10.1093/ofid/ofad500.2181

**Published:** 2023-11-27

**Authors:** Beomjin Shin, Eun Kyuong Chung, S Christian Cheatham, Megan R Fleming, Daniel P Healy, Michael B Kays

**Affiliations:** Kyung Hee University, Seoul, Seoul-t'ukpyolsi, Republic of Korea; Kyung Hee University, Seoul, Seoul-t'ukpyolsi, Republic of Korea; Antimicrobial Resistance Solutions, Carmel, Indiana; Eskenazi Health, Indianapolis, Indiana; University of Cincinnati, Cincinnati, Ohio; Purdue University, Indianapolis, Indiana

## Abstract

**Background:**

The objectives of this study were to describe population pharmacokinetics (PK) of cefepime and to evaluate pharmacodynamics (PD) of cefepime based on population PK model with various dosing regimens through Monte Carlo simulation to optimize dosing regimens for obese patients.

**Methods:**

Hospitalized adult patients who required antimicrobial therapy with cefepime for a suspected or documented bacterial infection were included in this study. Patients were stratified into either obese (body mass index [BMI] ≥ 30 kg/m^2^) or non-obese (BMI < 30 kg/m^2^) group. Based on serial concentration-time data in serum at steady state, population PK analysis was performed using NONMEM. Monte Carlo simulations were conducted in 6000 virtual patients using PK parameters of the final model for the following dosing regimens: 1 g q6h, 1 g q8h, 1 g q12h, 2 g q8h, and 2 g q12h. Probability of target attainment (PTA) for ≥ 60% free drug concentrations exceed the minimum inhibitory concentration (*f*T >MIC) was calculated at MICs ranging from 0.125 to 32 mg/L. Dosing regimens with PTAs ≥ 90% were considered optimal.

**Results:**

A total of 176 serum cefepime concentrations obtained from 24 patients (15 obese and 9 non-obese patients) were included in the population PK analysis. A one-compartment model best described the serum concentration-time data of cefepime. Fixed-exponent allometric scaling for volume of distribution with TBW significantly improved the model fit. Creatinine clearance was the only covariate significantly associated with cefepime PK, specifically systemic clearance (CL). Model-estimated V and half-life were significantly different between obese and non-obese patient groups (*P* < 0.01). At MICs ≤ 8 mg/L, all simulated intermittent infusions achieved PTA ≥ 90% in obese patients except 1 g q12h; only 2 g q8h regimen achieved PTA ≥ 90% in non-obese patients. For prolonged infusions, all tested dosing regimens achieved PTA > 90% except 1 g q12h in obese patients and q12h regimens in non-obese patients.

**Conclusion:**

Obesity significantly alters the PK of cefepime. However, it does not warrant alternative dosing in obesity. Prolonged infusions or larger doses may be considered at higher MICs or *f*T >MIC targets. Dosage adjustments based solely on obesity may be unnecessary.

**Disclosures:**

**All Authors**: No reported disclosures

